# The integrity of psychophysical visual function in non-immunocompromised PLHIV (NIPLHIV) without retinitis on antiretroviral therapy (ART)

**DOI:** 10.4314/ahs.v23i1.16

**Published:** 2023-03

**Authors:** Alvin Jeffrey Munsamy, Rune L Brautaset, Anandan A Moodley

**Affiliations:** 1 University of KwaZulu-Natal College of Health Sciences, Optometry; 2 Karolinska Institute, Clinical Neuroscience; 3 University of KwaZulu-Natal College of Health Sciences, Neurology

**Keywords:** HIV, vision disorders

## Abstract

**Purpose:**

The present study investigated the integrity of contrast sensitivity (CS), colour vision, and pattern evoked vision potentials (VEP) in non-immunocompromised people living with HIV (NIPLHIV) without retinitis.

**Methods:**

All participants were visually asymptomatic and no history of ocular disorders, with CD4 counts above 350 cells/mm^3^, low viral loads and on ART. Thirty NIPLHIV and 30 age-matched HIV negative control groups underwent F100 hue colour assessment, Pelli-Robson contrast sensitivity assessment and pattern-reversal VEP.

**Results:**

The median F100 total error scores for NIPLHIV and controls was 33 (IQR: 28;41) and 28 (IQR: 26;48.50) respectively, this was statistically different (p= 0.020). The median P100 amplitude for NIPLHIV was 5.75 µV (IQR: 4.4;8.85) and 4.05 µV (IQR: 3.2;5.8) for controls, this was statistically different (p=0.045). The mean LogCS score 1.83±0.14 and the median P100 peak latency was 105.45 msec (IQR: 102.98;108.98) for NIPLHIV. Higher CD4+ counts were significantly associated with having higher F100 total error scores (OR=0.995; p=0.018), lower P100 amplitudes (OR=1.007; p=0.010) and higher P100 latencies (OR=0.994; p=0.011).

**Conclusion:**

Contrast sensitivity function, colour vision, and VEP were uncompromised in NIPLHIV. Associations between CD4 counts with F100 total error scores and P100 latency may aid in the surveillance of vision of NIPLHIV.

## Introduction

The number of people with HIV (PLHIV) living normal lives (PLHIV) has increased with the universal access to antiretroviral therapy (ART). The reduction in viral loads and improved CD4+ counts has reduced episodes of retinitis previously observed in PLHIV with low CD4+ counts.^1^,^2^ However, the decrease in retinitis in PLHIV may not necessarily suggest that visual function is intact; or that subtle changes are not occurring. Visually asymptomatic PLHIV on ART, without retinitis, may or may not share the visual status of their HIV-negative counterparts with a similar immune status. There is evidence that shows compromised visual function, including contrast sensitivity (CS); visual evoked potentials (VEP) and electro-retinograms (ERG); colour vision and perimetry; in PLHIV on ART without retinitis, at CD4+ counts below 200 cells/mm^3^.^3^–^6^ However, there is limited evidence on functional visual integrity in PLHIV at higher CD4+ counts and lower viral loads (VL), owing to the improvement in access to ART, rendering present-day PLHIV as individuals with non-immuno-compromised status. Thus, assessing areas of visual function to quantify the differences, or lack thereof, in visually asymptomatic non-immunocompromised PLHIV on ART, without retinitis, in comparison to their HIV-negative counterparts, may be useful to practitioners in monitoring any threats to vision.

Previous studies^4^,^7^ assessing contrast sensitivity using the Pelli-Robson chart found that PLHIV without retinitis, on ART, with CD4+ counts below 200 cells/mm^3^, showed a median LogCS of 1.65, implying sub-normal changes. Shah et al.^3^ used a sample of PLHIV with a CD4+ count of 330 cells/mm^3^ and also found a median logCS of 1.65 when compared to HIV-negative persons. Demirkaya et al.^8^ Most recently studied HIV-infected men over 45 years of age on ART, with a median current CD4+ count of 595 cells/mm^3^ for at least 12 months and found a statistically significant difference equating to a one letter loss of logCS using the Pelli-Robson. A mean LogCS of 1.89 for the HIV group was obtained which, in comparison to the previous studies at lower CD4+ counts, is normal. The Pelli-Robson max score is LogCS 2.0, however scores greater than 1.65 are considered normal.

Studies^3^,^6^ assessing colour vision in PLHIV on ART without retinitis, using the Farnsworth-Munsel 100 Hue (FM100) test, observed higher total error scores (mean squares), suggesting reduced colour acuity. Shah et al.^3^ found a mean square root total error score of 10.20 in PLHIV with a CD4+ count of 330 cells/mm^3^; whilst Mueller et al.^6^ observed a score of 8.31 at a CD4+ count of 173 cells/mm^3^. The present study will attempt to address the gap in the research by studying colour vision in PLHIV on ART at higher CD4+ counts (> 330 cell/mm^3^) and lower viral loads.

Transient pattern reversal VEP was initially investigated by Iragui et al.^9^ In PLHIV on ART without retinitis and found delayed P100 latent periods (peak latency) at CD4+ counts below 200 cells/mm^3^, when compared to HIV-negative persons. The P100 peak latency for PLHIV on ART, with CD4+ counts less than 200 cells/mm^3^, was 109.06 msec, compared to those with counts greater than 200 cells/mm^3^, with a peak latency of 106.98 msec (p=0.007). This suggests that a delayed latent period is associated with a lower CD4+ count. Transient pattern reversal ERGs for the same sample also showed P100 amplitude differences with a reduced amplitude for PLHIV on ART. The lack of VEP studies at higher CD4+ counts is a gap in the literature worth investigating. Multifocal ERG studies^5^,^10^ also demonstrated reduced amplitudes and delayed latencies in PLHIV on ART, but at CD4+ counts less than or greater than 100 cells/mm^3^. The present study investigated contrast sensitivity, colour vision and pattern evoked potentials in visually asymptomatic NIPLHIV on ART, without retinitis, and compared them to age and sex-matched HIV-negative controls to assess for any changes that NIPLIV may not be aware of. Asymptomatic NIPLHIV referred to the visual status of the participants not suffering from compromised functional loss of vision or ocular health.

## Material and Methods

The study is observational and cross-sectional in design. All participants attending the outpatient clinics between August 2018 and November 2019 at Grey's tertiary hospital in Pietermaritzburg, KwaZulu-Natal, South Africa, were recruited using a purposive sampling technique^11^ due to the researcher assurance of non-immunocompromised status and ocular status of the participant to guard against confounders. Participants were given information sheets and, after written consent had been obtained, the following screening tests were conducted: Snellen visual acuity and non-cycloplegic auto-refraction; fundus photography; slit-lamp biomicroscopy; and a chart review, to obtain CD4+ counts, viral loads, the length of time infected with HIV, and any systemic history.

### Study Population

All patients attending the hospital outpatient clinics of a public hospital in South Africa. These patients were sourced from the surgical outpatient clinic for trauma, hernia and abdominal conditions. Patients who were HIV positive on ART who satisfied the selection criteria comprised the cases for the study whilst patients who were HIV negative presenting to the same clinics for similar surgical issues. All participants did not have infectious or inflammatory conditions for both cases and controls.

### Selection Criteria

All participants had to be over 18 years of age with CD4+ counts above 350 cells/mm^3^ and low- to- undetectable (less than 10000 copies/mL) viral loads, on ART for the previous six months. The study inclusion criteria were as follows: best-corrected visual acuity of 6/7.5 or better; no retinitis or history of ocular disease such as glaucoma, cataracts, uveitis or surgery; no systemic co-infections; and no strabismus or nystagmus. Any participant with refractive errors greater than 6 D myopia; 5 D hyperopia; 1.5 D astigmatism and 1.50 D anisometropia were excluded to guard against the influence of high refractive errors confounding any observations.

The study participants comprised PLHIV on ART (cases); and HIV-negative individuals (PLHIVN), who were the control group. After the inclusion criteria were satisfied, both groups underwent Farnsworth F100 hue colour assessment and Pelli-Robson contrast sensitivity assessment; but only the PLHIV went on further for assessment of pattern visual evoked potentials, due to hospital gatekeeper's demand for sick patient care, so access for the control group was not granted.

### Procedure

#### Farnsworth F100 Hue (Good-lite) Colour Vision Assessment

Previous studies^3^,^6^ used the FM100 Hue test to assess colour vision changes in PLHIV, employing the square-root of the total error score as an indicator of severity. Therefore, the present study utilised the F100 Hue test to assess the impact of immuno-competent HIV status on colour vision by calculating the total error score and its square-root as indicators to detect change. The procedure was as per test manual at good-lite.com^12^. The test was conducted at a comfortable working distance under standard room illumination for both eyes as instructed in the manual. The general time spent on each box was approximately two minutes. The error scores were calculated for each cap as the distance between any two caps. The total error score is the sum of all error scores for each cap. Weak colour discrimination was indicated by total error scores greater than 100, or a square root greater than 10; average colour discrimination was represented by total error scores between 16 and 100; whilst good colour discrimination was represented by total error scores of less than 16.^13^ Thereafter, blue-yellow (B-Y) and red-green (R-G) partial error scores were calculated.^14^ The partial error scores for the blue-yellow (B-Y) were then calculated using caps 1 through 12, 34 through 75, and 76 through 84. The partial error scores for the red-green (RG) axes were calculated using caps 13 through 33, and 55 through 75. All scores were calculated electronically using the Farnsworth F100 Score template at good-lite.com.

#### Pelli-Robson Contrast Sensitivity Assessment

Previous studies^49–51^ have demonstrated contrast sensitivity changes in immunocompromised PLHIV using the Pelli-Robson contrast sensitivity charts which utilises log scores. Therefore, in this study the contrast sensitivity assessment was conducted using the Pelli-Robson contrast chart to standardise the comparison. The chart has eight lines of letters with two groups of three letters (triplets) per line. Testing was performed at 1 m under standard consulting room lighting between 189 and 377 lux. The test was performed for monocular and binocular vision with best corrected prescription. The endpoint, or threshold log MAR contrast sensitivity, was recorded when the subject read the faintest triplet for which two of the three letters were named correctly. The logarithm to the base 10 of the contrast sensitivity measurement obtained was analysed for each sample for monocular measurements. LogCS of less than 1.5 were graded as abnormal, whilst scores less than 1.65 but greater than 1.5 were graded as a mild deficiency.^3^,^4^,^7^

#### Pattern-Reversal Visual Evoked Potential (VEP) measurements (Nihon Kohden Neuropack M1)

Participants were seated at a distance of 1.5 m from the visual stimulus wearing the appropriate refraction equipment (corrected for the test distance). The (active) recording electrode was placed on the posterior scalp and joined to the positive input of the differential amplifier. A reference electrode was placed on a visually neutral site on the head, such as the earlobe, and was connected to the negative terminus. A ground electrode was placed on the forehead. Responses to about 200 stimuli (100–300) were amplified and averaged for each eye, which were then analysed with an automatic artefact rejection software. Monocular recordings were performed separately for the left and right eyes. A minimum of two trials for each eye were usually procured and then superimposed for ensuring replicability of the VEP pattern. The P100 amplitudes and peak latency for each eye were analysed to study the integrity of the visual pathway.

### Data Analysis

#### Data and variables for analysis

Variables representing psychophysical visual function is/are the data obtained from F100 colour vision test, Pelli-Robson CS test and the P100 VEP tests. These were the total and partial error scores; LogCS scores and P100 peak latencies and amplitudes. The logistic regression also included age; duration of ART; CD4 counts and CD4 percentage and viral loads.

### Statistical analysis

**Figure 3:**
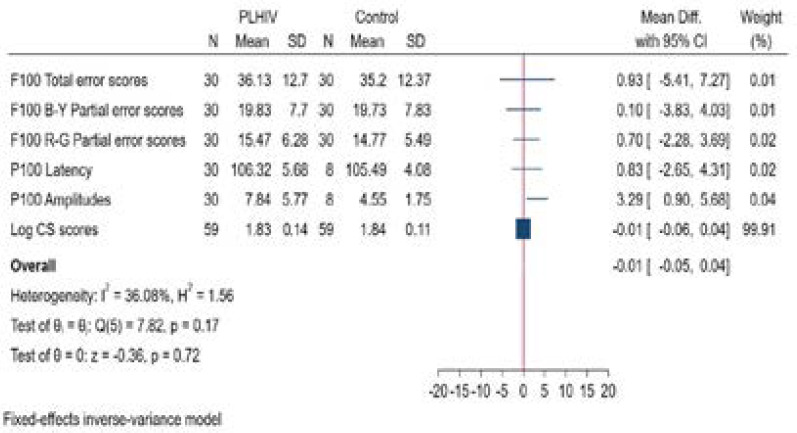
Forest plot of the effect size of observations for visual function between PLHIV and the control group

A statistician carried out all analysis using Stata version 16 software. Descriptive statistics were used to compare the NIPLHIV on ART (cases) with the age- and sex-matched HIV-negative control group using the independent t-test and the Mann-Whitney test at a significance level of p <0.05. Where applicable, medians and interquartile ratios and box and whisker plots were presented to account for skewness of data. The mean differences were included and shown as forest plot (Figure 3.) to illustrate the magnitude or size of difference (mean difference) between groups, considering the statistical significance. The Cohen's d statistic was then calculated to standardise the mean differences and establish the effect size on the difference of the means of the two groups. Finally, logistic regression with *p* value less than 0.05 was used to identify the determinant factors associated with visual function. To further visualize the multivariable association of covariates and visual function, forest plots were applied. All the tests were two-tailed and the criterion for statistical significance was set at 5% level.

### Ethical considerations

Ethical permission was obtained from the Biomedical Research and Ethics Committee (ethical clearance reference number: BE 359/17) at the University of Kwa-Zulu-Natal. All participants signed an informed consent prior to commencement of the study. All researchers ensured that participant information was kept confidential. The conduct of the study complied with the Declaration of Helsinki regarding research on human subjects.

## Results

### Study participants

**Figure 1:**
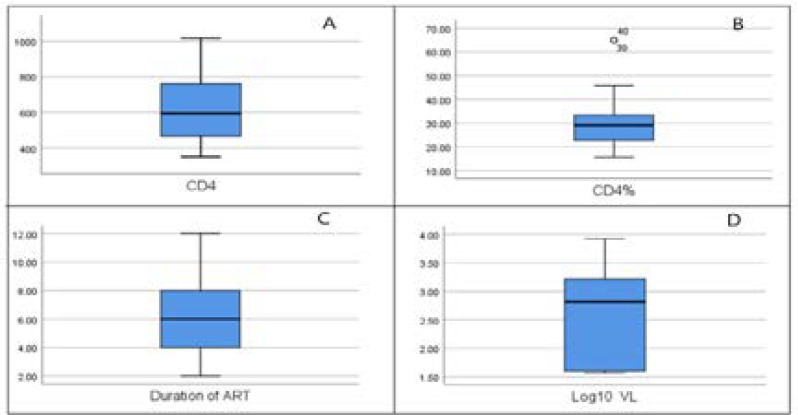
Box and whisker plots for indices of immunocompetence for PLHIV (A) CD4+count (cell/mm^3^), (B) CD4 percentage, (C) Duration of ART (years), (D) Viral Load (copies/ml)

Thirty (N=30) participants (58 eyes) comprised the HIV-positive group and 30 (58 eyes) participants who were age- and sex-matched comprised the HIV-negative control group. The median age for the PLHIV was 39.50 years (IQR: 33; 45); and 39 years (IQR: 30.75; 44) for the control group. Each group had 13 male and 17 female participants. The PLHIV had a median CD4+ count of 594 cells/mm^3^ (IQR: 460; 771); a median CD4% of 28.57% (IQR: 22.59-33.15); a median viral load of 0 copies/ml (IQR: 0-46.25) and a median time on ART of six years (IQR: 4; 8). These indices of immunocompetence are presented in the Box and whisker plots in Figure 1.

**Figure 2:**
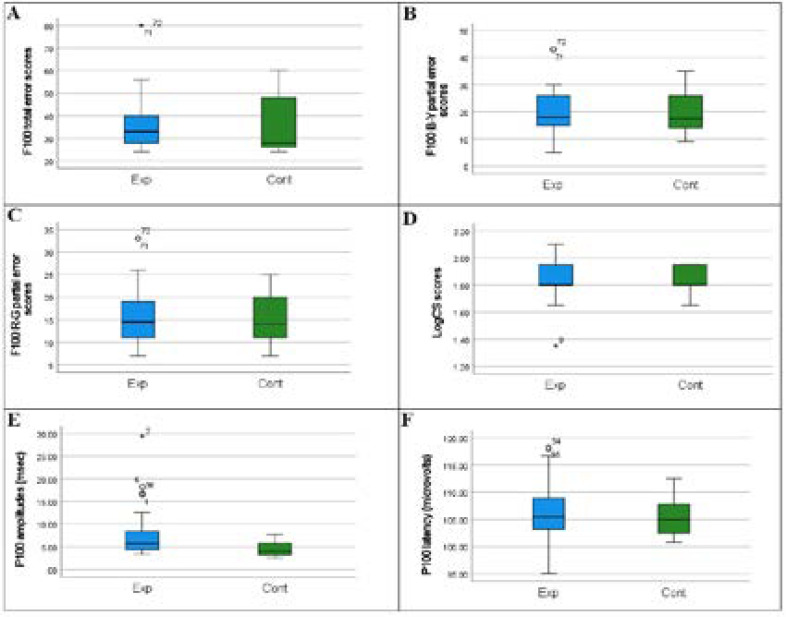
Box and whisker plots of F100 total error scores (A), F100 B-Y partial error scores (B), F100 R-G partial error scores (C), LogCS scores (D), P100 amplitudes (msec) (E) and P100 latency (microvolts) for both experimental (Exp) and control (Cont) groups

Figure 2 shows the box and whisker plots of the comparisons of visual function between the control and experimental groups.

The frequency of abnormal contrast sensitivity function (CS) (<1.5 log units) was found in 3.4% of eyes in NIPLHIV on ART; whilst there was none in the control group, however no statistically significant differences (p=0.15) between the two groups were observed. Figure 2D shows a complementary box and whisker plot showing the median LogCS of 1.80 [IQR:1.80-1.95] for NIPLHIV. The frequency of mild CS dysfunction (<1.65 log units) was identified in 16.9% of eyes in NIPLHIV on ART and 16.7% of eyes in the control group, however, no statistically significant differences (p=0.97) between the two groups were observed. The frequency of weak colour vision dysfunction, defined as a total error score greater than 100, occurred in none of the participants, in either the PLHIV-on-ART or control groups. All participants had average total error scores (16-100).

### Contrast sensitivity in non-immunocompromised PLHIV

The mean LogCS score for PLHIV and healthy age-matched controls was 1.83±0.14 and 1.84±0.11 respectively as shown in Table 1. Analysis showed (See Table 1) that there was no statistically significant difference between NIPLHIV on ART and the age- and sex-matched controls, for the Pelli-Robson scores (p=0.636). The mean difference calculation was -0.01 for LogCS scores which is illustrated in Figure 3. The Cohen's d effect size was then calculated to be -0.08 [95%CI: -0.44,0.28] which is regarded as small (<0.2). For our study this implies that this observation has limited practical significance.

**Table 1:** Visual function results in PLHIV on ART and controls

Visual Function	N (eyes)	Controls				N (eyes)	PLHIV				P-value
		Mean ±SD	Median [IQR]	Skewness	Kurtosis		Mean ±SD	Median [IQR]	Skewness	Kurtosis	
**Contrast sensitivity**											
Log CS scores	30 (59)	1.84±0.11	1.80 [1.8–1.95]	-0.534	-1.045	30 (59)	1.83±0.14	1.80 [1.80–1.95]	-0.854	1.815	0.636^a^
**Colour Vision**										
F100 Total error score	30	35.2±12.26	28.0 [26.0–48.5]	0.866	-0.827	30	36.13±12.59	33.0 [28.0–41.0]	1.724	3.236	**0.020** b
F100 Total error score (RMS)	30	5.85±0.99	5.3 [5.1–6.9]			30	5.93±0.96	5.7 [5.3–6.3]			0.252^a^
F100 B-Y partial error score	30	19.73±7.76	17.5 [13.7–26.0]	0.612	-0.854	30	19.83±7.64	18.0 [14.7–26.0]	0.720	1.240	0.772^a^
F100 B-Y partial error score (RMS)	30	4.36±0.86	4.2 [3.7–5.1]			30	4.37±0.86	4.24 [3.9–5.1]			0.681^a^
F100 R-G Partial error score	30	14.77±5.44	14.0 [10.5–20.0]	0.386	-0.917	30	15.47±6.23	14.50 [11.0–19.5]	0.856	0.446	0.749^a^
F100 R-G Partial error score (RMS)	30	3.78±0.71	3.4 [3.3–4.5]			30	3.86±0.77	3.8 [3.3–4.4]			0.650^a^
**Visual Evoked** **Potentials**											
P100 Amplitude (µV)	4(8)	4.55±1.75	4.05[3.2–5.8]	0.79	-0.25	4(8)	7.84±5.77	5.7 [4.4–8.8]	2.285	5.951	**0.045** b
P100 Peak latency (msec)	4(8)	105.49±4.08	105[102.4–107.8]	0.79	-0.23	4(8)	106.32±5.68	105.4 [103.0–109.0]	0.374	0.207	0.720^a^

**CS**: Contrast Sensitivity, **RMS**: Root mean square

a Independent t-test

b Mann-Whitney-U-test

### Colour vision in non-immunocompromised PLHIV

Table 1 shows the median F100 total error scores for NIPLHIV and healthy age-matched controls was 33 (IQR: 28;41) and 28 (IQR: 26;48.50) respectively also shown in Fig. 2A as a box and whisker plot. It can also be seen that the F100 Hue test also showed a statistically significant difference in total error scores (p = 0.020). The effect size calculation was 0.93 for the F100 hue total error scores. Thus, the mean difference between the two groups was large, as is illustrated in Figure 3. The median root mean square scores were 5.74 (IQR: 5.29;6.32) for NIPLHIV and 5.29 (IQR: 5.10;6.92) for healthy age-matched controls and were shown to not be significantly different (p=0.252).

Table 1. shows the median B-Y partial error score was 18 (IQR: 14.75;26) for NIPLHIV and 17.50 (IQR: 13.75;26) for healthy age-matched controls. Fig. 2B illustrates this as a box and whisker plot. Table 1 shows the median R-G partial error score was 14.50 (IQR: 11.0-19.5) for NIPLHIV and 14.0 (IQR: 10.5-20.0) for healthy age-matched controls. Fig. 2C illustrates this as a box and whisker plot. Table 1 also shows that there was no statistically significant difference for partial error scores between both groups. However, as shown in Figure 3, the mean difference for R-G partial error scores was 0.7. However, the Cohen's d effect size was then calculated to be 0.12 [95%CI: -0.39,0.6], which is regarded as small effect size (<0.2) and may have limited practical significance for our study.

### Visual evoked potentials in non-immunocompromised PLHIV

Table 1 and Fig. 2E and 2F shows the median P100 amplitude for NIPLHIV was 5.75 µV (IQR: 4.4;8.85) and 4.05 µV (IQR: 3.2;5.8) for healthy age-matched controls. Table 1 shows a statistically significant difference between the NIPLHIV and the age-matched HIV control group for P100 amplitudes (p=0.045). The mean difference was 3.29 as is illustrated in Figure 3. The Cohen's d effect size was then calculated to be 0.63 [95%CI: -0.16,1.42] which is regarded as moderate (>0.5). For our study this implies that this observation may have practical significance. Table 1 shows the median P100 peak latency was 105.45 msec (IQR: 102.98;108.98) for NIPLHIV and 105 msec (IQR: 102.45;107.85) for healthy age-matched controls. No significant difference was noted for P100 peak latency between the groups (p=0.72). The mean difference as shown in Figure 3 was 0.83. The Cohen's d effect size was then calculated to be 0.15 [95%CI: -0.63,0.93] which is regarded as small (<0.2) and may thus have limited practical clinical significance for our study.

### Associations between indices of immunocompetence and visual function in non-immunocompromised PLHIV

**Figure 4:**
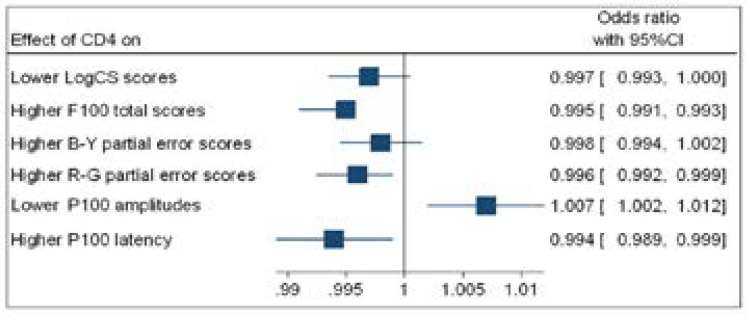
Forest plot (binary logistic regression outputs) showing odds ratio (OR) for higher CD4+count and visual function

**Figure 5:**
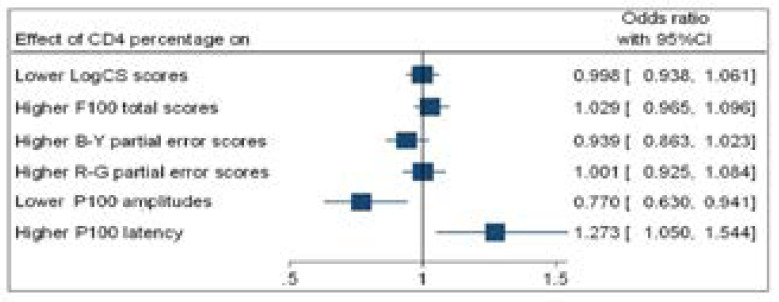
Forest (binary logistic regression outputs) plot showing odds ratio (OR) for higher CD4 percentage and visual function

**Figure 6:**
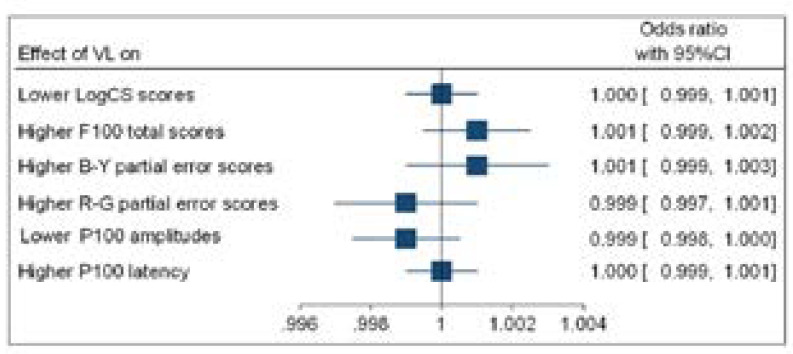
Forest (binary logistic regression outputs) plot showing odds ratio (OR) for higher viral loads (VL) and visual function

**Figure 7:**
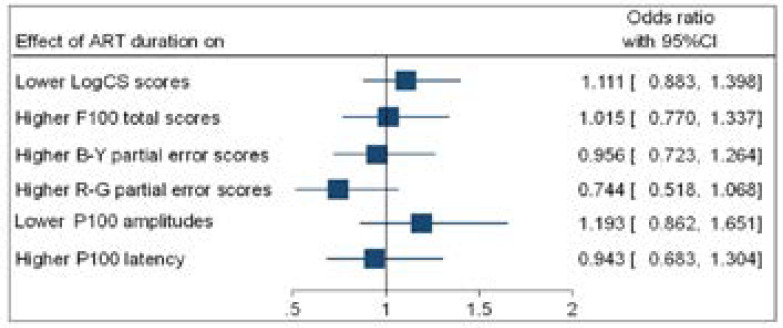
Forest plot (binary logistic regression outputs) showing odds ratio (OR) for higher duration of ART and visual function

The results of a binary logistic regression between F100 scores; P100 latency; P100 amplitudes, LogCS scores and CD4+ counts; CD4 percentages; viral load and time (duration) on antiretroviral therapy for non-immunocompromised people living with HIV are shown in Figures 4 to 7. The associations are represented by the odds ratio (OR).

Figure 4 shows that participants with higher CD4+ counts were significantly associated with higher F100 total error scores (OR=0.995; *p*=0.018); higher F100 R-G partial error scores (OR=0.996; *p*= 0.048); lower P100 amplitudes (OR=1.007; *p*=0.010); and higher P100 latencies (OR=0.994; *p*=0.011).

Figure 5 shows that higher CD4 percentages were only significantly associated with lower P100 amplitudes (OR=0.777; *p*=0.011) and higher P100 latencies (OR=1.27; *p*=0.014). No significant associations were observed for lower CS scores and higher F100 error scores.

Figures 6 and 7 show that higher a viral load (VL) and more time on ART had no significant association with lower CS scores; higher F100 error scores; lower P100 amplitudes and longer P100 latencies.

## Discussion

The study endeavoured to compare the visual function of a sample of non-immuno-compromised PLHIV on ART, without retinitis, who were visually asymptomatic, with age- and sex-matched HIV-negative persons, using a cross-sectional approach, and specifically observing contrast sensitivity function, colour vision and VEP. There were only statistically significant differences in one aspect of visual function (i.e. F100 total error scores) between the sample of NIPLHIV on ART and the HIV negative sample. Furthermore, the prevalence of abnormal CS, colour vision and P100 peak latency did not differ between the two samples. However, using binary logistic regression, the study was able to establish significant associations between CD4+ counts and percentages and F100 total error scores and P100 amplitudes and latency.

The most recent evidence on CS in PLHIV with high CD4+ counts and no retinitis by Demirkaya et al.^8^ found LogCS of 1.89, which is similar to the present finding's LogCS of 1.80. These log scores were still higher than those for PLHIV on ART, without retinitis, at lower CD4+ counts. Our study was unable to establish a relationship with LogCS scores and CD4+ counts and viral loads.

F100 total error scores showed a significant difference between the groups, with PLHIV having weaker scores, which also showed large effect sizes, suggesting possible practical implications. Shah et al.^3^ Assessed colour vision in PLHIV on ART, without retinitis, at CD4+ counts of 300 cells/mm^3^, using the FM100 hue test, and found a mean total error score (root mean square) of 10.20. This may be comparable to the present study's finding of 5.94 when considering the error score range and the higher current median CD4+ count of 594 cells/mm^3^. No other studies have examined partial error scores for B-Y and R-G, which did not show any significant differences between the groups. However, the R-G partial error scores showed a moderate effect size that may be an emerging area of concern. A noteworthy observation was that higher CD4 counts have a reduced chance of higher F100 total error scores and higher F100 R-G partial error scores, suggesting that a reduction in CD4+ counts will reduce colour discrimination; or that maintaining higher CD4+ counts prevent the deterioration of colour vision. This suggests, indirectly, that ophthalmic practitioners who track any drop in total error scores, without clinical colour vision deficits, could alert PLHIV to their possible declining immune status as immunocompromised individuals. This facility in LMIC countries with resource-stricken health systems, which only deal with PLHIV in clinics, may complement the care of PLHIV by the eye-care fraternity.

The present study also found that the pattern reversal P100 peak latency of 106 msec at a CD4+ count of 594 cells/mm^3^ is comparable to that of Iragui et al.^9^, who also found 106 msec, despite a lower CD4+ count of 200 cells/mm^3^. Both studies used a sample of PLHIV on ART without retinitis and both results lie within the normal range of 95 to 114 msec, according to the present study's laboratory. Although P100 amplitudes were significantly different between the groups, this observation had a large effect size, which may translate into clinical differences. However, the smaller sample size in the control group may confound this observation. The present study's findings of 7.84 µV, which was lower than the normal 10.3 µV, may also require confirmation in future studies in non-immunocompromised PLHIV, considering the wide range of P100 amplitudes in the present study. However, it may be regarded normal when factoring in the standard deviations. The P100 amplitude difference (3.29 µV) noted may require an increased sample size of the control group before it can be conclusive, in light of all the other variables being insignificantly different. However, there is a paucity of studies on P100 amplitudes in NIPLHIV. Higher CD4+ counts have an increased likelihood of larger P100 amplitudes and a decreased likelihood of longer P100 latencies, suggesting that reduced CD4+ counts will compromise VEP. Thus, the logistic regression showed that maintaining higher CD4+ counts will reduce the likelihood of further delays in P100 latency. Higher CD4 percentages have a decreased chance of lower P100 amplitudes and an increased chance of longer P100 latencies, suggesting that reducing CD4 percentages may also compromise VEP. CD4 percentages are not a routine indicator of immunity, accept for children with hyperactive immunity, and its use here was purely as a secondary indicator of stable immune status in PLHIV with elevated CD4+ counts. This emphasises the singular role ART may play in the maintenance of visual function in this sample of NIPLHIV, considering their elevated CD4+ counts and reduced viral loads.

## Limitations

The study findings will require confirmation in future studies as the sample size prohibits generalisation to the broader population of non-immuno-compromised NIPLHIV on ART. The absence of randomization may also introduce bias as well they may also limit generalisations. The P100 amplitudes shown in Fig. 2E, has considerable outliers which may be attributed to the small sample size. However, its value lies in the paucity of studies of PLHIV with higher CD4+ counts, larger sample sizes and demographic variations. The effect size calculations did not factor in sample size, but did show a small effect in the mean difference in observations between the two samples, for those comparisons that were not significant but may complement the independent t-test's statistically insignificant difference between the two groups. Another limitation is the lack of longitudinal observation, and treatment with ARTs for six years, in the present sample. The significance of this result lies in the possibility that visual status changes as the duration of ART use increases, and as the type of therapy changes. Closely following the cohort over time would strengthen this observation. However, the study's median viral load of zero, implying undetectable viral loads, may complement the elevated CD4+ counts of this sample. This suggests that, should changes in ART occur, any discrepancies found in the future (providing the viral loads remain undetectable) may not be related to the virus but the therapy. Present-day ART has been associated with adverse systemic effects15, including metabolic and nervous system involvement, which already have general ocular associations.

Furthermore, considering that CD4+ counts fluctuate daily, using a single observation may be a limiting factor. However, the high CD4+ counts of the sample were supported with the recording of a mean CD4 percentage of 28.57%, which is above 25%, which for PLHIV allows for complementary indicators of a non-immuno-compromised status.

The choice of the tests for visual function of Pelli-Robson; F100 Hue and VEP are not routine clinical investigations and were chosen due to the visually asymptomatic status of the sample populations. Standard clinical evaluations may not have been sensitive enough to detect sub-clinical changes. Considering this choice, the observations about visual function may validate the choice of the Pelli-Robson CS test and VEP, and may infer the integrity of the visual pathway in this sample of non-immunocompromised PLHIV beyond near-normal visual acuity. This suggests that the undisturbed integrity of visual function in non-immunocompromised NIPLHIV may be verified by future studies, especially considering the paucity of work in this area.

## Conclusion

This investigation revealed non-compromised contrast sensitivity function, colour vision and pattern reversal VEP in PLHIV on ART with elevated CD4+ counts and reduced viral loads. Although the study findings may not be generalised, they may help practitioners benchmark visually asymptomatic PLHIV with extended lifespans, and without retinitis, should this population become symptomatic. The significance of the results may contribute to the scarce literature in this field of HIV and vision until such time as a large-scale prospective study is completed. The monitoring of these visual functions for deterioration from baseline may aid in the surveillance of non-immunocompromised PLHIV with extended lifespans, afforded by ART, as evidenced by the associations between CD4+ counts and F100 total and partial error scores, and P100 amplitudes and latency.
